# Risk of neuropsychiatric and cardiovascular adverse events following treatment with varenicline and nicotine replacement therapy in the UK Clinical Practice Research Datalink: a case–cross‐over study

**DOI:** 10.1111/add.15338

**Published:** 2020-12-14

**Authors:** Kyla H. Thomas, Neil M. Davies, Amy E. Taylor, Gemma M. J. Taylor, David Gunnell, Richard M. Martin, Ian Douglas

**Affiliations:** ^1^ Bristol Medical School, Population Health Sciences University of Bristol Bristol UK; ^2^ Medical Research Council Integrative Epidemiology Unit University of Bristol Bristol UK; ^3^ K.G. Jebsen Center for Genetic Epidemiology, Department of Public Health and Nursing, NTNU Norwegian University of Science and Technology Norway; ^4^ National Institute for Health Research, Bristol Biomedical Research Centre University Hospitals Bristol NHS Foundation Trust and University of Bristol Bristol UK; ^5^ Addiction and Mental Health Group (AIM), Department of Psychology University of Bath Bath UK; ^6^ Department of Non‐communicable Disease Epidemiology, Faculty of Epidemiology and Population Health LSHTM London UK

**Keywords:** Adverse events, cardiovascular, neuropsychiatric, nicotine replacement therapy, observational study, varenicline

## Abstract

**Background and aims:**

Varenicline and nicotine replacement therapy (NRT) are the most commonly used medications to quit smoking. Given their widespread use, monitoring adverse risks remains important. This study aimed to estimate the neuropsychiatric and cardiovascular risks associated with varenicline and NRT as used in routine UK care.

**Design:**

Case–cross‐over study.

**Setting:**

UK‐based electronic primary care records in the Clinical Practice Research Datalink from 2006 to 2015 linked to hospital and mortality data sets.

**Participants:**

Adult smokers (*n* =282,429) observed during periods when exposed and not exposed to either varenicline or NRT.

**Measurements:**

Main outcomes included suicide, self‐harm, myocardial infarction (MI), all‐cause death and cause‐specific death [MI, chronic obstructive pulmonary disease (COPD)]. In primary analyses, conditional logistic regression was used to compare the chance of varenicline or NRT exposure during the risk period (90 days prior to the event) with the chance of exposure during an earlier single reference period (91–180 days prior to the event) or multiple 90‐day reference periods to increase statistical power.

**Findings:**

In the primary analyses, findings were inconclusive for the associations between varenicline and the main outcomes using a single reference period, while NRT was associated with MI [odds ratio (OR) = 1.40, 95% confidence interval (CI) = 1.18–1.67]. Using multiple reference periods, varenicline was associated with an increased risk of self‐harm (OR = 1.32, 95% CI = 1.12–1.56) and suicide (OR = 3.56, 95% CI = 1.32–9.60) but a reduction in all‐cause death (OR = 0.75, 95% CI = 0.61–0.93). NRT was associated with MI (OR = 1.54, 95% CI = 1.36–1.74), self‐harm (OR = 1.30, 95% CI = 1.18–1.44) and deaths from MI (OR = 1.53, 95% CI = 1.11–2.10), COPD (OR = 1.33, 95% CI = 1.14–1.56) and all causes (OR = 1.28, 95% CI = 1.18–1.40) when using multiple reference periods.

**Conclusions:**

There appear to be positive associations between (1) nicotine replacement therapy (NRT) and myocardial infarction, death and risk of self‐harm and (2) varenicline and increased risk of self‐harm and suicide, as well as a negative association between varenicline and all‐cause death. The associations may not be causal. They may reflect health changes at the time of smoking cessation (nicotine replacement therapy is prescribed for people with cardiac problems) or be associated with quit attempts (exposure to both medicines was associated with self‐harm).

## Introduction

Smoking is the leading preventable cause of morbidity and mortality in many countries [1, 2]. Varenicline, bupropion and nicotine replacement therapy (NRT) are all licensed as smoking cessation medicines in the United Kingdom; however, bupropion is much less commonly prescribed than other medications [3]. Varenicline is the most effective smoking cessation medicine in monotherapy; a network meta‐analysis of randomized controlled trials showed that for every 10 smokers who quit with single‐form nicotine replacement therapy (NRT) or bupropion, approximately 16 would be expected to quit with varenicline [4]. Consistent findings were reported in a large prospective cohort study which showed that patients prescribed varenicline were more likely to be smoking‐abstinent than those prescribed NRT, an association which persisted for up to 4 years [5]. However, varenicline has not been shown to be more effective than combination NRT (for example, nicotine patch plus a faster‐acting form of NRT such as nasal spray, gum or inhalator [4].

Concerns regarding the cardiovascular and neuropsychiatric safety of varenicline led regulatory agencies to issue safety warnings about varenicline's possible adverse effects [6, 7]. From 2009 to 2016, the US Food and Drug Administration (FDA) required that varenicline carry a Black Box warning on its product labelling; this is the agency's strongest safety warning [6]. Although the Black Box warning was removed by the FDA in December 2016 [8], concerns about varenicline persist among some. Coroners have linked varenicline to several suicides in Australia; the FDA's decision to downgrade the safety warning has also been criticized [9]. Concerns have also been raised previously concerning the relationship between NRT and serious cardiovascular adverse events in older studies [10, 11]. These findings have not been supported by a recent Cochrane Review, which found little evidence that NRT increased the risk of MI, although it increased the odds of chest pains and palpitations relative to control [12].

Various study designs with differing strengths and limitations [13] have been used to investigate these safety issues, including case reports, observational cohort studies and meta‐analyses. Whereas studies using data from spontaneous reporting systems have reported an increase in psychiatric adverse effects such as suicide with varenicline use [14], large observational studies, randomized controlled trials (RCTs), meta‐analyses and network meta‐analyses of RCTs have not supported these findings [4, 15, 16, 17, 18, 19, 20, 21, 22, 23, 24]. Additionally, large meta‐analyses have provided conflicting evidence regarding whether patients prescribed varenicline are at increased risk of adverse cardiovascular events such as myocardial infarction [25, 26, 27, 28]. Similarly, there are conflicting reports regarding the cardiovascular safety of NRT. A meta‐analysis by Mills *et al*. found that NRT was associated with an elevated risk of chest pain and heart palpitations [29]. However, their more recent network meta‐analysis found no evidence that NRT was associated with major adverse cardiovascular events, although an elevated risk was observed for all cardiovascular events, including less serious events such as heart palpitations [28]. A 2018 Cochrane Review reported similar findings [12].

There are concerns regarding the validity of findings using different study designs. First, although RCTs are considered the gold standard for the evaluation of the intended effects of medicines, they are rarely powered or designed to detect rare unintended adverse effects. Although one of the key aims of meta‐analyses is to combine data from multiple trials and, in effect, increase the sample size, the sample size requirements for rare outcomes, e.g. suicide, may still be prohibitively large [30]. Secondly, although observational pharmacoepidemiological studies that utilize large primary care databases are more likely to meet the sample size requirements for identifying rare adverse outcomes, they are prone to residual or uncontrolled confounding, in particular confounding by indication. Confounding by indication may arise because individuals who are prescribed a particular medication are likely to differ from those who are not prescribed the drug, because there is a reason or indication for prescribing a drug [31]. For example, the use of smoking cessation medicines may appear to be associated with an increased risk of cardiovascular disease. However, smoking itself is a major risk factor for cardiovascular disease. One approach to overcoming confounding by indication is to compare rates of adverse events in patients prescribed different drugs to treat the same underlying condition (i.e. use of active comparators) [32].

Epidemiological study designs which rely only on cases, known as case‐only designs, are increasingly used to avoid pitfalls such as confounding and selection bias, which may occur in observational studies with control groups such as cohort and case–control studies [33]. Case‐only designs (which include the case–cross‐over method, case–time control method and self‐controlled case–series) may benefit from the elimination of time‐invariant within‐person confounding factors such as socio‐economic position and genetic predisposition. Other benefits include having greater statistical power to detect rare adverse effects and being less costly to carry out compared with conventional observational studies [33].

In the current study, we estimate the neuropsychiatric and cardiovascular adverse risks of varenicline and NRT in the UK Clinical Practice Research Datalink (CPRD) using a case–cross‐over study design.

## Methods

### Study design and patients

**Figure 1:**
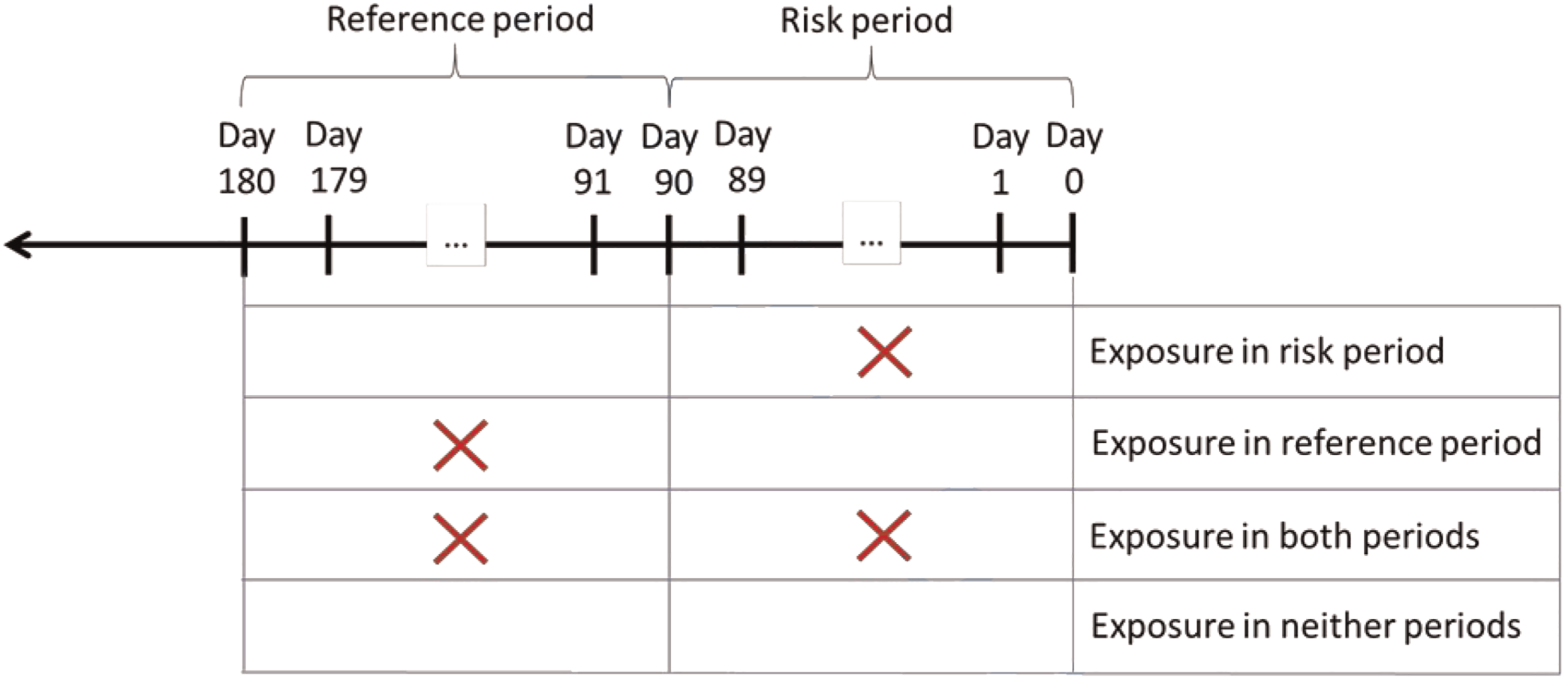
Case–cross‐over analysis illustrating risk and reference periods and exposure to treatment. ‘X' represents exposure to a particular treatment. Concordance occurs where there is exposure to treatment in both periods or exposure in neither periods. Discordance occurs where there is exposure to treatment in the risk period but not the reference period or exposure in the reference period but not the risk period

The CPRD is one of the largest primary care databases in the world and contains electronic medical records from > 15 million individuals, who are representative of the UK population [34]. In the United Kingdom > 98% of the population are registered with a general practitioner (GP), who act as gatekeepers of care for the National Health Service. Data from GP consultations as well as information which is fed back from secondary care referrals are routinely entered onto computers, creating the electronic medical records of which the CPRD is comprised. We used data from the CPRD and linked hospital admissions data from the Hospital Episode Statistics (HES) database and mortality data from the Office of National Statistics (ONS) mortality data set to conduct a population‐based case–cross‐over study. The case–cross‐over method is a type of case‐only design which is epidemiologically and statistically comparable to matched case–control analyses, except the case serves as his/her own control [35, 36, 37]. In the simplest design, study participants are compared at two different time‐points (see Fig. 1); the first time‐point is nearer to the occurrence of the event of interest (referred to as the risk period); the second‐time point represents a similar time‐interval occurring further away from and earlier than the event of interest (referred to as the reference period). Therefore, if a particular treatment were actually associated with a specific outcome, it would be expected that exposure to that treatment would occur more frequently during the risk period than the reference period. The similarity of the case–cross‐over study to the matched case–control design occurs as only discordant pairs (i.e. those exposed during the risk period but not the reference period and vice versa) contribute to the statistical analysis. Individuals with concordant matched pairs (i.e. exposed or unexposed to treatment during both time‐periods) are uninformative.

All hypotheses and analyses (with the exception of the analyses exploring time‐dependent confounding) were pre‐specified in a study protocol which was approved by the Independent Scientific Advisory Committee (ISAC) (http://research‐information.bristol.ac.uk/en/persons/kyla‐h‐thomas(e3917519‐6a48‐4192‐af81‐a1199d545b40)/projects.html; last accessed 18 March 2020). We used the most recent version of CPRD Gold available at the time (November 2015).

### Participants

Patients were included if they were adult smokers from 1 September 2006 (when varenicline was licensed in the United Kingdom) onwards to 31 November 2015. Smokers were defined as patients who have a smoking record which indicates current smoker (obtained from the ‘Additional Clinical Details’ file in the CPRD) or Read codes, which indicate current smoking after 1 September 2006. Read codes are a coded thesaurus of clinical terms which are used in electronic health‐care records in the UK National Health Service. Read code algorithms to define smoking status were based on those used in a previous study by Szatkowski & McNeill in The Health Improvement Network (THIN) database, which is similar to the CPRD [38]. The prevalence of current smoking identified from primary care electronic health records has previously been shown to accurately reflect the prevalence reported in national surveys, such as the Health Survey for England [39].

Records from patients classified as ‘acceptable’ by the CPRD from all up‐to‐standard practices at least 18 months prior to date of entry of each cohort (1 January 2005) were included. Patient data were defined as ‘acceptable’ by the CPRD if they met minimum quality control standards; for example, they had information on sex, date of birth and first registration with no breaks in registration, i.e. a valid GP registration period. Up‐to‐standard practices included those which reported when their patients first registered with the practice and left the practice, with continuous data reporting in between.

Patients were excluded if they were registered at a GP practice for fewer than 365 days before the first recorded prescription. We excluded patients prescribed both NRT and varenicline at the same time. In a previous CPRD analysis, this occurred for 0.25% of all prescriptions [18].

### Exposures, outcomes and covariates

Cases included smokers who had experienced one of the following smoking‐related outcomes: suicide, non‐fatal self‐harm (suicide attempt), myocardial infarction (MI) and death from all causes and the following specific causes: MI, lung cancer and chronic obstructive pulmonary disease (COPD) (the latter were included as major causes of smoking‐related morbidity and mortality). CPRD Read codes were used to identify self‐harm and MI using validated algorithms [40, 41]. HES data were used to identify inpatient hospital admissions for self‐harm. Deaths were identified using ONS mortality data. We used linked ONS mortality data to identify MI deaths, as previous research has shown that failure to do so may result in biased estimates of MI incidence and outcome [41]. Similarly, CPRD recording of suicide has also been shown to be unreliable, although the under‐reporting of self‐harm is less marked [40]. The following International Classification of Disease Tenth Revision (ICD‐10) codes were used for mortality: MI (codes I21–I22), COPD (codes J40–J44), lung cancer (C34, C78, D02.2, D14.3, D38.1), suicide (intentional self‐harm, codes X60–X84) and events of undetermined attempt, codes Y10–Y34). In England and Wales, the ONS definition of suicides includes deaths given an underlying cause of intentional self‐harm, in addition to deaths caused by injury or poisoning where the intent was undetermined for those aged 15 years and over. This is because most undetermined deaths are likely to be suicides [42]. Inpatient self‐harm admissions were identified using the same ICD‐10 codes that were used to identify suicide deaths. Only incident events were included in the statistical analysis. Events were assumed to be independent.

Exposure to varenicline or NRT in the CPRD was identified using product codes. A product code is a unique code in the CPRD which is used to identify each specific prescribed medicine selected by a GP for treatment. Product codes are available from the ‘Therapy file’ of the CPRD.

### Statistical analysis

#### Primary analysis

For the primary analysis, the risk period was defined as 90 days prior to a specific outcome, while the reference period was defined as 91–180 days prior to the outcome. A time‐period of 90 days was chosen as the risk period, as the maximum recommended treatment duration for varenicline is 12 weeks (3 months) (https://bnf.nice.org.uk/drug/varenicline.html; last accessed 18 March 2020). NRT treatment for smoking cessation should also continue for up to 3 months before dose reduction (https://bnf.nice.org.uk/drug/nicotine.html#indicationsAndDoses; last accessed 18 March 2020). If a study participant was exposed to a particular smoking cessation medicine for at least 1 day in a given reference or risk period, the person was considered exposed to that medicine for the entire duration of that period. All analyses were repeated replacing exposure to varenicline with exposure to NRT. NRT was used as a comparator, as its mechanism of action is different from varenicline; the association of both medicines with a specific adverse event could therefore imply the event was associated with the timing of smoking cessation instead of a causal effect of the medication. While the case–cross‐over method deals with time‐invariant confounding, time‐varying confounding remains a problem which this approach could potentially address indirectly.

Each study participant formed two halves of a matched pair, comparing exposure to varenicline during the risk period (90 days prior to the outcome event) with exposure to varenicline during a single reference period (90 days before the risk period). Conditional logistic regression was used to calculate odds ratios (ORs) and 95% confidence intervals (CIs) for the discordant matched pairs using the clogit command. Analyses were carried out using Stata statistical software version 14MP.

#### Secondary (sensitivity) analyses

Sensitivity analyses were repeated 30 and 180 days prior to the event as the risk period, such that the reference periods were 31–60 days prior to the event and 181–360 days prior to the event.

#### Multiple reference periods

Multiple reference periods were used to increase the statistical power of the primary and secondary analyses. This involved using up to a maximum of four reference periods compared to one risk period. For example, in the primary analysis, exposure to varenicline during the risk period (90 days prior to the event) was compared with exposure to varenicline during four 90‐day reference periods (i.e. 91–180, 181–270, 271–360 and 361–450 days prior to the event).

#### Assessment of time‐dependent confounding

Case–cross‐over designs assume no unmeasured time‐dependent confounding. We investigated the possibility of time‐dependent confounding in a *post‐hoc* exploratory analysis by estimating the rates of four events: primary care diagnoses and hospitalization for myocardial infarction and self‐harm. Primary care diagnoses were identified using Read codes in the CPRD. Hospital admissions were identified using the linked hospital admissions data set using the previously described ICD‐10 codes for self‐harm and MI. We performed this by extracting the weekly number of records indicating each of the four events during the year before and the year after the patients were prescribed any NRT or varenicline prescription. This means that there are multiple prescriptions per person and the denominator for this analysis is all NRT or varenicline prescriptions. We set week zero to be the week before the index prescription. We then plotted the event rate by dividing the number of events per week by the number of NRT and varenicline prescriptions.

##### Data statement

Data used in the project are available from a third party, the Clinical Practice Research Datalink (contact info enquiries@cprd.com). The data can be accessed by submitting an application to the Independent Scientific Advisory Committee (https://cprd.com/Data‐access). Ethical approval was not required for this project.

## Results

The baseline characteristics (median age and sex) of participants experiencing events (excluding lung cancer) are shown in Table 1. A flow‐chart of the number of patients and prescriptions assessed for eligibility and the reasons for exclusion is presented in Supporting information, Fig. S1. The number of events for each outcome is shown in Table 2. Lung cancer deaths were excluded from further analysis due to the very small number of events identified. For the majority of patients dying from lung cancer, NRT was not prescribed during either the risk or the reference periods; for varenicline this was the case for all lung cancer deaths. NRT was prescribed during the reference period but not the risk period for < 5 lung cancer deaths.

**Table 1:** Baseline characteristics of the cases included in the analyses (people experiencing events).

Characteristic	Outcomes under investigation						
	Myocardial infarction events	Myocardial infarction deaths	Self‐Harm events	Self‐Harm Hospital Admissions	Suicide deaths	COPD deaths	All deaths
All	19 664	3461	25 455	12 584	679	8730	51 786
% female	30.9	36.4	55.5	54.7	25	44.8	44.2
Median age in years	65	75	36	37	45	77	75

Abbreviation: COPD = chronic obstructive pulmonary disease.

**Table 2:** OR and 95% CIs of exposure to varenicline and NRT using 90‐day risk and reference periods for specific adverse events.

Adverse event	Number of events	Number exposed risk period but not exposed reference period	Number not exposed risk period but exposed reference period	OR (95% CI) 1 : 1 matching	OR (95% CI) 1 : 4^a^ matching
Varenicline					
MI events	19 664	96	113	0.85 (0.65–1.12)	1.19 (0.98–1.45)
Self‐harm events	25 455	151	141	1.07 (0.85–1.35)	**1.32 (1.12–1.56)**
Self‐harm hospital admissions	12 584	57	66	0.86 (0.61–1.23)	1.08 (0.83–1.42)
MI deaths	3461	8	10	0.80 (0.32–2.03)	0.82 (0.44–1.66)
Suicide deaths	679	7	2	3.50 (0.73–16.85)	**3.56 (1.32–9.60)**
COPD deaths	8730	24	26	0.92 (0.53–1.61)	0.92 (0.64–1.37)
All cause deaths	51 786	84	105	0.80 (0.60–1.07)	**0.75 (0.61–0.93)**
NRT					
MI events	19 664	303	216	**1.40 (1.18–1.67)**	**1.54 (1.36–1.74)**
Self‐harm events	25 455	433	414	1.04 (0.91–1.20)	**1.30 (1.18–1.44)**
Self‐harm hospital admissions	12 584	155	183	0.85 (0.68–1.05)	1.08 (0.92–1.26)
MI deaths	3461	36	32	1.13 (0.70–1.81)	**1.53 (1.11–2.10)**
Suicide deaths	679	11	7	1.57 (0.61–4.05)	1.32 (0.69–2.53)
COPD deaths	8730	155	146	1.06 (0.85–1.34)	**1.33 (1.14–1.56)**
All‐cause deaths	51 786	556	533	1.04 (0.93–1.18)	**1.28 (1.18–1.40)**

^a^ Matching on a maximum of four 90‐day reference (reference) periods to increase statistical power. Non‐null findings are shown in bold type. MI = myocardial infarction; OR = odds ratio; CI = confidence interval; NRT = nicotine replacement therapy; COPD = chronic obstructive pulmonary disease.

Table 2 also shows the association between adverse events in smokers and exposure to varenicline or NRT using 90‐day risk, and up to a maximum of four reference periods.

### Single reference period

For a single 90‐day risk period compared to the immediately preceding 90‐day reference period there was inconclusive evidence that varenicline was associated with an increased risk of self‐harm (OR = 1.07, 95% CI = 0.85–1.35); while the risk of suicide was elevated, estimates were imprecise and confidence intervals spanned the null value (OR = 3.50, 95% CI = 0.73–16.85). There was inconclusive evidence of an association between varenicline and self‐harm hospital admissions (OR = 0.86, 95% CI = 0.61–1.23), deaths from MI (OR = 0.80, 95% CI = 0.32–2.03) or COPD (OR = 0.92, 95% CI = 0.53–1.61]). There was a positive association between NRT and MI (OR = 1.40, 95% CI = 1.18–1.67), with inconclusive evidence for other outcomes.

### Multiple reference periods

When multiple 90‐day reference periods were used with a single 90‐day risk period to increase statistical power, there was evidence that varenicline was associated with an increased risk of self‐harm (OR = 1.32, 95% CI = 1.12–1.56) and a more than threefold increased risk of suicide (OR = 3.56, 95% CI = 1.32–9.60). However, varenicline was associated with a reduction in deaths from all causes (OR = 0.75, 95% CI = 0.61–0.93). NRT was associated with an increased risk of MI (OR = 1.54, 95% CI = 1.36–1.74), self‐harm (OR =1.30, 95% CI = 1.18–1.44), MI deaths (OR = 1.53, 95% CI = 1.11–2.10), COPD deaths (OR = 1.33, 95% CI = 1.14–1.56) and all‐cause deaths (OR = 1.28, 95% CI = 1.18–1.40). There was inconclusive evidence for an association of NRT with suicide (OR = 1.32, 95% CI = 0.69–2.53) or self‐harm hospital admissions (OR = 1.08, 95% CI = 0.92–1.26).

### Sensitivity analyses

Secondary (sensitivity) analyses using 30‐ and 180‐day risk and reference periods are shown in Supporting information, Tables S2 and Table S3, respectively, and were largely consistent with the findings of the multiple reference period analyses. Using a 30‐day risk and reference period, varenicline was associated with a reduced risk of all‐cause mortality. NRT was associated with an increased risk of MI. For the 180‐day risk and reference periods, varenicline was associated with a reduction in all‐cause mortality and COPD deaths and an increased risk of MI, self‐harm and inpatient self‐harm admissions (using multiple reference periods only). NRT was associated with an increased risk of MI and self‐harm. However, NRT was also associated with an increase in MI deaths and all‐cause mortality (using multiple reference periods).

**Figure 2:**
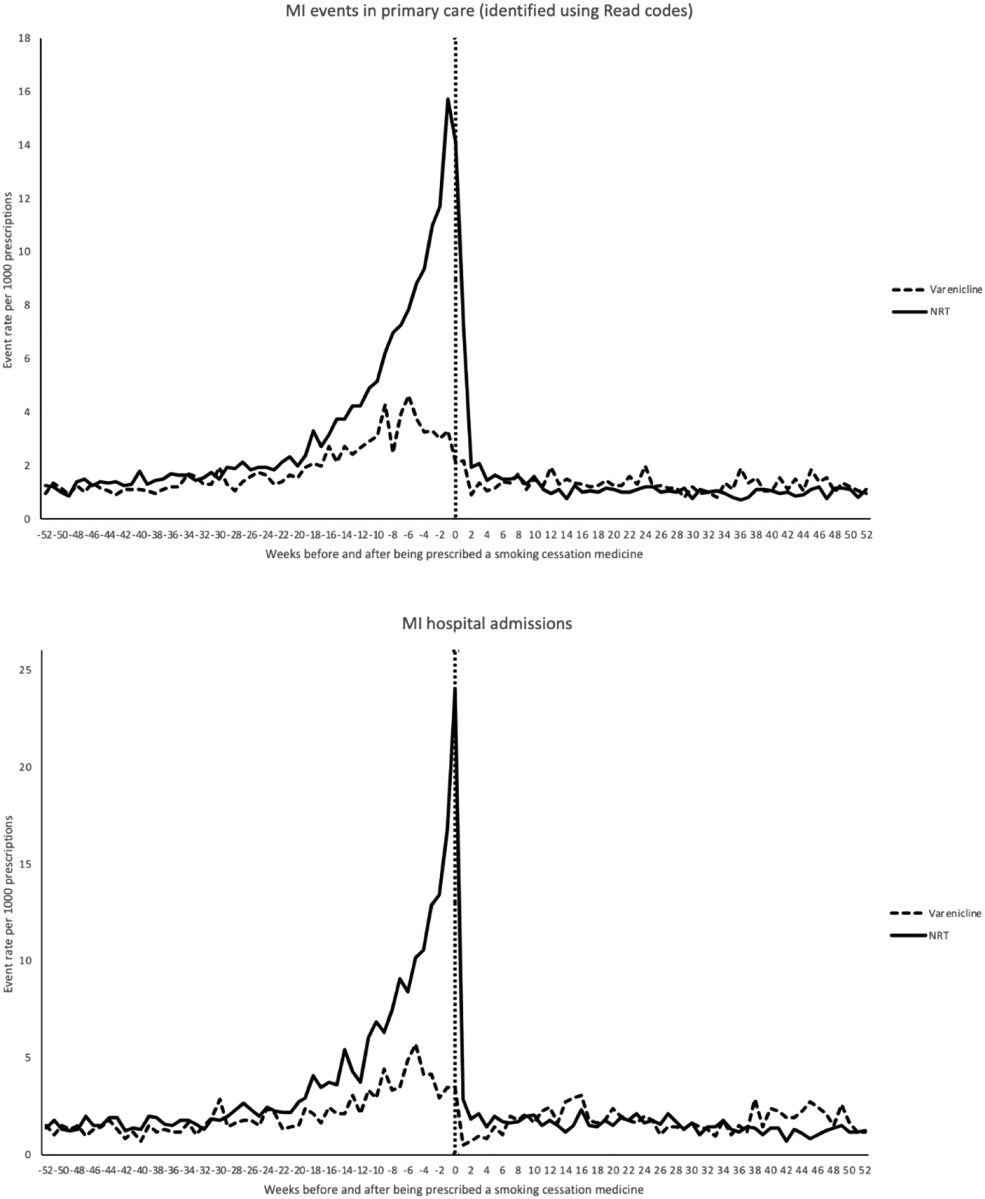
Rate of myocardial infarction (MI) events and hospital admissions per 1000 prescriptions in the weeks before and after being prescribed varenicline or nicotine replacement therapy

Figure 2 illustrates the rate of primary care diagnoses of and hospital admissions for myocardial infarction during the 52 weeks before and after varenicline and NRT prescriptions. Negative values on the *x*‐axis indicate the weeks before the prescription, positive values indicate the weeks after the prescription. There was a significant increase in the number of diagnoses of MI events during the weeks leading up to a NRT prescription (from 1.2 MI events per 1000 prescriptions 52 weeks before being prescribed NRT to 15.7 events per 1000 prescriptions during the week before being prescribed NRT), followed by a very substantial fall in the number of diagnoses during the weeks following a prescription (from 14.1 events per 1000 during the week of being prescribed NRT to between 1 and 1.5 events per 1000 from the fourth week after being prescribed NRT onwards). The results were similar for the relationship between hospital admissions for myocardial infarction and NRT prescribing. A similar temporal trend was observed with varenicline prescriptions, although it was much less marked. These findings may be due to non‐fatal cardiovascular events or symptoms triggering prescriptions; in our analyses, prescription of a smoking cessation product is likely to be affected by within‐individual time‐dependent confounding.

**Figure 3:**
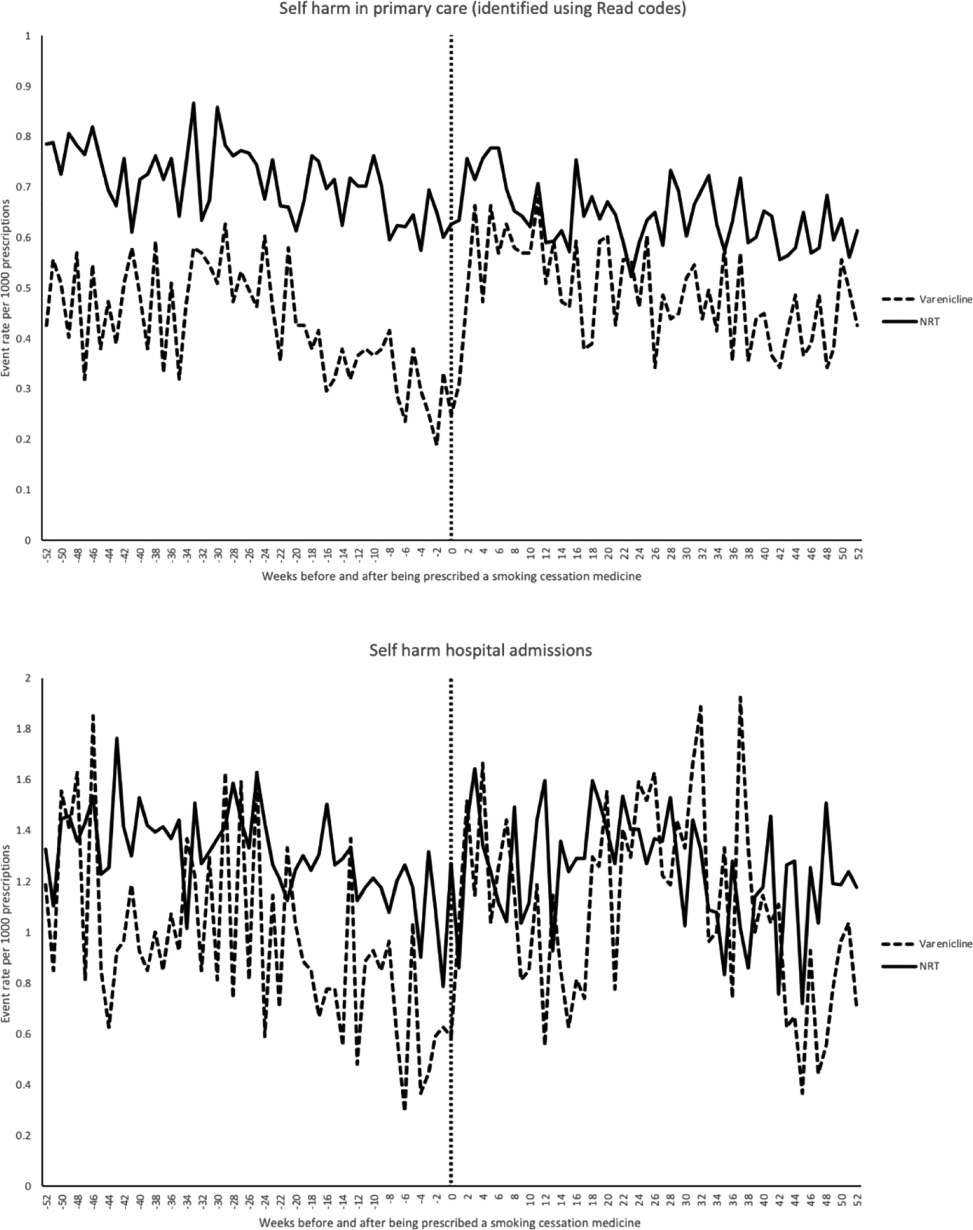
Rate of self‐harm events and hospital admissions per 1000 prescriptions in the weeks before and after being prescribed varenicline or nicotine replacement therapy

Figure 3 illustrates the event rates per 1000 prescriptions for primary care diagnosis and hospital admissions for self‐harm. There were much smaller changes in the event rate per 1000 prescriptions for self‐harm events compared with MI events over time. Overall, there were small changes in the self‐harm event rates before and after NRT prescriptions were issued (event rates were consistently between 0.6 and 0.7 per 1000 prescriptions). However, self‐harm events per 1000 prescriptions were markedly lower during the weeks before a varenicline prescription (0.1–0.2 events per 1000) compared with the weeks following a varenicline prescription (0.3–0.6 events per 1000), showing that varenicline was less likely to be issued if the patient had a recent primary care diagnosis of self‐harm, consistent with prescribing guidelines. Similar findings were observed for self‐harm hospital admissions.

## Discussion

### Main findings

In the primary analysis using a single 90‐day risk period and reference period we found inconclusive evidence that varenicline increased the risk of any of our outcomes of interest. Although NRT was associated with a 40% (95% CI = 18–67%) increased risk of MI there was strong evidence of time‐dependent confounding, suggesting that MI (or heart disease more generally) may lead to the prescription of NRT. Findings were also sensitive to design decisions. When multiple 90‐day reference periods were used to increase statistical power, varenicline was associated with a 256% (95% CI = 32–860%) increased risk of suicide, 32% increased risk of self‐harm and a 25% reduction in all‐cause mortality. Similarly, NRT was associated with an increased risk of self‐harm and deaths from all‐causes, MI and COPD. There was inconclusive evidence of an increased risk of self‐harm hospital admissions with varenicline or NRT. In the secondary analyses, varenicline was associated with a reduction in all‐cause deaths using the shorter 30‐day time window for the risk and reference periods and NRT was associated with an increased risk of MI. However, using multiple reference periods and the 180‐day risk and reference periods, positive associations were observed for MI and self‐harm (varenicline and NRT), self‐harm hospital admissions (varenicline only) and deaths from MI and all causes (NRT only).

### Strengths and limitations

The use of data from the CPRD is one of the main strengths of this study. Study participants are likely to be more representative of patients prescribed smoking cessation medicines in the United Kingdom compared with the highly selected patients usually included in randomized controlled trials. Secondly, we used validated code lists and linked data sets to improve the accuracy of detection of our outcomes of interest [40, 41]. Thirdly, we used the case–cross‐over method to investigate the association of varenicline and NRT with adverse outcomes. Advantages of this study design include its ability to completely control for between‐person confounding, minimizing within‐person time‐invariant confounding factors (i.e. subject characteristics that remain constant) and statistical efficiency (the use of multiple reference periods for one risk period increases statistical power) [35]. Also, as we investigated varenicline as well as NRT, we could assess whether events may have resulted from nicotine withdrawal (e.g. the increased risk of self‐harm events observed with both treatments during the 90‐day risk and reference period when multiple reference periods were used).

A major study limitation is the observational study design. Therefore, the analysis was still prone to residual time‐variant confounding, in particular within‐person confounding by transient factors; for example, changes in disease severity or comorbid conditions [37]. The result of within‐person comparisons would also be affected by the choice of comparison periods. We observed strong time‐dependent confounding, shown by the temporal patterns in the occurrence of MI and self‐harm‐related events before and after smoking cessation medication prescribing in the exploratory analyses. Patients were more likely to be prescribed NRT following a primary care diagnosis of MI and hospitalization for MI. Patients prescribed varenicline were less likely to have had a primary care diagnosis or hospital admission for self‐harm during the weeks prior to the prescription. This may be because GPs were less likely to prescribe varenicline to patients who have recently self‐harmed. Although we observed an association between both varenicline and NRT and self‐harm events in our primary analyses using multiple reference periods, we did not find evidence of any associations with self‐harm hospitalizations. This may have been caused by a lack of statistical power, as we identified half as many self‐harm hospitalizations as self‐harm events.

We were unable to perform case–time control analyses as stated in our original protocol as we could not obtain a sufficient number of matched controls. This would have allowed statistical adjustments to be made for a common time trend, such as a change in the prescribing pattern of the smoking cessation medicines [37]. However, this is unlikely to be an issue during the short time‐periods utilized in the main analyses. It is important to note that our analyses were also sensitive to some of our design decisions; for example, the number of matching periods and the duration of the risk and reference periods. In the primary analysis, the use of multiple reference periods provided a point estimate in a more harmful direction to the result using a single reference period for MI and self‐harm hospital admissions in the varenicline group. Additionally, for both varenicline and NRT, increases in the length of the risk and reference periods from 30 to 180 days resulted in a greater number of positive associations using multiple reference periods. This may be indicative of a temporal bias which was not fully accounted for in the analyses; i.e. with increasing time from the event occurrence, the potential for time‐dependent confounding increases due to changes in the individual such as changes in health status. This is suggested by the strong temporal pattern of event rates we observed around the time smoking cessation medication was started.

Our analyses were also restricted to products prescribed in primary care (thus excluding patients receiving smoking cessation products in smoking cessation clinics or buying over‐the‐counter NRT from pharmacies). Those who visit a health‐care professional for prescribed medications are likely to be sicker and to be less affluent or of a lower socio‐economic position compared to those buying over the counter medicines [43]. Therefore, the analyses may not be generalizable to the wider population of people taking smoking cessation medicines, including those obtained over the counter without a prescription. Additionally, being prescribed medication does not mean that the patient actually took the medication. We had no information on treatment compliance or adherence, but problems with either would tend to bias results towards a null effect.

### Comparison with other case‐only studies

Three recent studies have used within‐person designs to investigate the neuropsychiatric and cardiovascular safety of varenicline [44, 45, 46]. Monarrez‐Espino *et al*. carried out a case–cross‐over study using data from Swedish health and administrative registers [44]. They reported on four different hazard (risk) periods, including a hazard period of 1–84 days, which approximates to our main analyses using a 90‐day risk and reference period. There was inconclusive evidence that varenicline was associated with MI (OR = 0.98, 95% CI = 0.80–1.22), suicide (OR = 0.58, 95% CI = 0.32–1.06) or suicide attempt (OR = 0.82, 95% CI = 0.63–1.07). However, varenicline was associated with a reduction in the outcome which combined suicide and suicide attempt (OR = 0.77, 95% CI = 0.60–0.98). These findings are not consistent with our study, possibly due to differences in the study populations or differences in prescribing behaviour for smoking cessation in Sweden compared with the United Kingdom, leading to different temporally associated changes in risk. Gershon *et al*. used a self‐controlled risk interval study design to investigate neuropsychiatric and cardiovascular hospitalizations with varenicline [45]. Similar to the case–cross‐over study, each patient acts as his/her own control, minimizing within‐person time‐invariant confounding. However, it differs from the case–cross‐over study design, as for patients exposed to a particular treatment it examines the risk of the outcome of interest during a specified period closest to the exposure (risk period) with a remaining observation period (control period). For new users of varenicline, the authors found a 34% higher incidence of cardiovascular events during the 12‐week risk period compared with the control interval (relative incidence = 1.34, 95% CI = 1.25–1.44). An increase in the incidence of neuropsychiatric events was also observed for varenicline (relative incidence = 1.06, 95% CI = 1.00–1.13). This finding is similar to our finding for the association of varenicline and self‐harm in the main analyses (OR = 1.07, 95% CI = 0.85–1.35). The differences in the results for cardiovascular outcomes may be due to the differences in estimation of the risk periods and population size. The authors did not examine outcomes in relation to NRT.

Molero *et al*. used a within‐person comparison cohort design to examine associations between varenicline and a range of outcomes including new psychiatric conditions and suicidal behaviour [46]. Although varenicline was not shown to be associated with suicidal behaviour [hazard ratio (HR) = 1.00, 95% CI 0.72–1.37], it was associated with an increase in the risk of anxiety conditions (HR = 1.27, 95% CI = 1.06–1.51) and mood conditions (HR= 1.28, 95% CI = 1.07–1.52). Suicidal behaviour was defined as emergency in‐ or outpatient hospital visits or death due to intentional self‐harm and differed from our analyses, as they did not include ICD codes for undetermined events or deaths.

One study examined the use of NRT and the risk of acute MI, stroke and death in the THIN, using the self‐controlled case series method [47]. The incidence of MI increased during the 56 days prior to the first prescription of NRT [incidence ratio (IR) = 5.55, 95% CI = 4.42–6.98], although it was not increased during the 56 days following the first NRT prescription (IR = 1.27, 95% CI = 0.82–1.97). However, there was an increased risk of MI during the first 14 days following NRT prescription (IR = 2.39, 95% CI = 1.28–4.48), which is consistent with our findings.

### Comparison with other study designs

With respect to neuropsychiatric outcomes, our results from the primary analyses using a single 90‐day risk period and multiple 90‐day reference periods are consistent with prescription event monitoring studies and studies using adverse event reporting databases, which have reported an increased risk of reported suicidal behaviour for varenicline compared with NRT [14, 48, 49, 50, 51]. However, previous studies which included comparison groups (i.e. RCTs, meta‐analyses of RCTs and other observational study designs) have reported inconclusive findings as to whether varenicline is associated with an increased risk of suicide, suicide attempt or other mental disorders (depression, neurotic disorders or prescriptions for anti‐depressants) [15, 16, 17, 18, 19, 20, 21, 22]. This could partly be because most RCTs and meta‐analyses of RCTs would not have sufficient statistical power to detect an effect of prescribing varenicline on such a rare outcome [19, 21]. For example, the large Evaluating Adverse Events in a Global Smoking Cessation (EAGLES) study found no significant increase in neuropsychiatric events with varenicline compared to placebo or NRT [21]. The study had a sample size of 8144 participants among four treatment groups; it was statistically powered to detect an adverse event which occurred in at least 4% of patients in any treatment group (a moderate effect size). However, a sample size of 21 584 would be needed for a clinical trial to detect the more than threefold increase we observed for suicide in this study, based on a suicide incidence rate of 9.2 per 100 000 at 80% power and 5% significance. Previous meta‐analyses of neuropsychiatric events have included < 12 000 participants and reported very few suicides; therefore, the lack of statistical power to detect an effect would also be an issue in these studies [19, 23]. Previous observational cohort studies which found inconclusive evidence between smoking cessation medicines and neuropsychiatric outcomes or a negative association were also likely to be impacted by residual confounding (those prescribed varenicline were healthier than those prescribed NRT) and/or the very limited numbers of suicides identified (< 0) [15, 18, 20, 24]. Our study found an association between self‐harm and being prescribed NRT or varenicline, which may be explained by an association between quit attempts and self‐harm. Although nicotine withdrawal is known to be associated with mood changes [52], evidence showing a clear association with self‐harm is lacking.

Our findings for all‐cause mortality suggest caution is needed when interpreting results. Varenicline was associated with a reduction in all‐cause mortality, consistent with findings using conventional methods of analyses (multivariable regression and propensity score matching) from previous UK primary care observational studies using the CPRD and the Q Research database [18, 20]. The protective effect of varenicline on all‐cause mortality was not driven solely by a reduction in COPD or MI deaths. However, we were unable to identify the specific causes behind this protective effect, as our CPRD extract did not include causes of death we had not prespecified in our protocol. Conversely, we found that NRT was associated with higher all‐cause mortality in our primary analyses using a single 90‐day risk period and multiple 90‐day reference periods. However, it is possible that all the analyses may have been affected by time‐dependent residual confounding. Additionally, previous studies have shown that people prescribed varenicline are likely to be healthier than those prescribed NRT [15, 18, 20].

Findings regarding the cardiovascular safety of varenicline are also conflicting. In this study, varenicline was only associated with an increased risk of MI events for the 180‐day risk and reference period using multiple reference periods. Although a 19% increased risk of MI events was observed during the 90‐day risk and reference periods, the 95% CI included the null. Previous studies (including the EAGLES study and its non‐treatment extension [53], meta‐analyses of RCTs [26, 28] and an observational study [20]) found no increase in cardiovascular events with varenicline or NRT. However, a systematic review of varenicline versus placebo found evidence of an increased risk [25]. The Mills *et al*. (network meta‐analysis also found an elevated risk of cardiovascular events associated with NRT, mainly due to less serious events, but was underpowered to assess the risk of serious events [28]. A recent cohort study using the CPRD also found an increase in cardiovascular events by 52 weeks for patients prescribed NRT compared with those receiving smoking cessation advice only [54]. These findings are consistent with our study. This association may be due to smokers who experience worsening of symptoms, such as chest pain, being more likely to seek help from their GPs to quit smoking (as shown by Fig. 2).

## Conclusions

In this study, we used a case–cross‐over study design to investigate the risk of neuropsychiatric and cardiovascular outcomes associated with varenicline and NRT in a real‐world setting. For primary analyses using a 90‐day risk period and multiple reference periods, we observed associations between varenicline and suicide and self‐harm as well as associations between NRT and self‐harm, MI, MI deaths and all‐cause mortality. However, these temporal associations may not be causal, as we also found strong evidence of time‐dependent confounding, particularly for our NRT analyses where those experiencing MI were likely to be prescribed NRT during the week before the event. The evidence was much less marked for varenicline. The association of both varenicline and NRT with self‐harm in our study may reflect an association between self‐harm and quit attempts, rather than a causal association with the smoking cessation medications. Additionally, associations such as a reduction in all‐cause mortality with varenicline and an increased risk of COPD deaths with NRT may be explained by differences in GP prescribing behaviour (healthier patients are prescribed varenicline) or changes in health status (for example, COPD exacerbation triggering NRT prescribing). Further evidence will be provided when the results of the largest network meta‐analysis of smoking cessation medicines and e‐cigarettes are reported [55]. The study will report on smoking abstinence in addition to safety outcomes, including serious adverse events, major adverse neuropsychiatric events (including suicide and self‐harm) and major adverse cardiovascular events. Further research can aim to replicate our study using similar data sets; for example, Scandinavian record linkage studies and large North American health‐care databases. Additionally, mendelian randomization and genetic correlation studies may provide further information on associations with self‐harm. What is clear is that, regardless of cause, people attempting to stop smoking with smoking cessation therapies appear to have a higher risk of neuropsychiatric and cardiorespiratory events which may be due to time‐dependent confounding (people who are sicker seeking treatment), or theoretically an effect of taking smoking cessation therapy. More research is needed to elucidate these relationships.

## Declaration of interests

All authors have completed the ICMJE uniform disclosure form at www.icmje.org/coi_disclosure.pdf and declare: K.H.T. received funding for the project from the Academy of Medical Sciences Starter Grant for Clinical Lecturers Scheme (supported by the Wellcome Trust, British Heart Foundation, Medical Research Council, Versus Arthritis, Prostate Cancer UK and the Royal College of Physicians). A.T. has received a Global Research Award for Nicotine Dependence (GRAND), an independently reviewed, competitive grants programme supported by Pfizer, to the University of Bristol. I.J.D. is supported by an unrestricted grant from GlaxoSmithKline and holds shares in GlaxoSmithKline. K.H.T. is currently funded by a National Institute for Health Research Postdoctoral Fellowship (PDF‐2017‐10‐068). Part of this work was undertaken while K.H.T. was funded by a clinical lectureship from the National Institute for Health Research. N.M.D. is supported by a Norwegian Research Council Grant Number 295989. G.T. is funded by a Cancer Research UK Population Researcher Postdoctoral Fellowship award (reference: C56067/A21330). I.J.D. is funded by an unrestricted grant from GlaxoSmithKline. R.M.M., D.G. and A.T. are supported by the NIHR Bristol Biomedical Research Centre, a partnership between the University Hospitals Bristol NHS Foundation Trust and University of Bristol. R.M.M. is also supported by a Cancer Research UK programme grant (C18281/A19169). G.T. and N.D. have received a Global Research Award for Nicotine Dependence (GRAND), an independently reviewed, competitive grants programme supported by Pfizer, to the University of Bristol.

## Author contributions

**Kyla Thomas:** Conceptualization; formal analysis; funding acquisition; project administration. **Neil Davies:** Conceptualization; formal analysis. **David Gunnell:** Funding acquisition. **Ian Douglas:** Conceptualization.

## Supporting information


**Figure S1** Flow chart showing the number of patients and prescriptions assessed for eligibility and reasons for exclusion.

**Table S2** Odds ratios and 95% confidence intervals of exposure to varenicline and NRT using 30‐day risk and reference periods for specific adverse events.

**Table S3** Odds ratios and 95% confidence intervals of exposure to varenicline and NRT using 180‐day risk and reference periods for specific adverse events.
