# Supplementary material for: Risk of neuropsychiatric and cardiovascular adverse events following treatment with varenicline and nicotine replacement therapy in the UK Clinical Practice Research Datalink: a case–cross‐over study
Source: Addiction. 2020 Dec 14;116(6):1532–45. doi: 10.1111/add.15338 (PMC8246946; doi:10.1111/add.15338)
Supplement: Supplementary file 1 — Figure S1 Flow chart showing the number of patients and prescriptions assessed for eligibility and reasons for exclusion. [file ADD-116-1532-s001.docx]

**Figure S1. Flow chart showing the number of patients and prescriptions assessed for eligibility and reasons for exclusion**

**
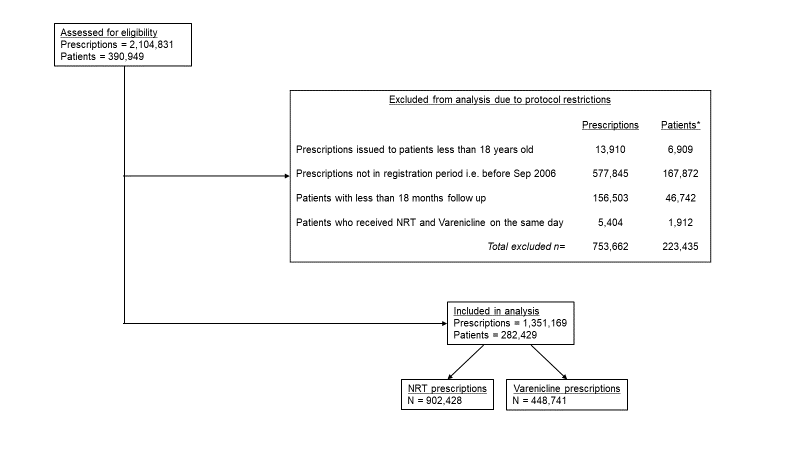
**

**390,949 patients were identified as smokers between 1^st^ September 2006 and 31^st^ November 2015. *As each patient can have multiple prescriptions, the sum of the number of patients excluded and the number of patients included will not be equal to the number of patients assessed for eligibility.**
