# Supplementary material for: Risk of neuropsychiatric and cardiovascular adverse events following treatment with varenicline and nicotine replacement therapy in the UK Clinical Practice Research Datalink: a case–cross‐over study
Source: Addiction. 2020 Dec 14;116(6):1532–45. doi: 10.1111/add.15338 (PMC8246946; doi:10.1111/add.15338)
Supplement: Supplementary file 2 — Table S2 Odds ratios and 95% confidence intervals of exposure to varenicline and NRT using 30‐day risk and reference periods for specific adverse events. [file ADD-116-1532-s002.docx]

**Table S2. Odds ratios and 95% confidence intervals of exposure to varenicline and NRT using 30-day risk and reference periods for specific adverse events.**

| Adverse event | Number of events | Number Exposed risk period  Not exposed ref period | Number Not exposed risk period Exposed ref period | OR (95% CI)  1:1 matching | OR (95% CI)  1:4 matching* |
| --- | --- | --- | --- | --- | --- |
|  |  | **Varenicline** |  |  |  |
| MI events | 20,599 | 35 | 45 | 0.78 (0.50-1.21) | 0.81 (0.59-1.12) |
| Self-Harm events | 27,076 | 52 | 57 | 0.91 (0.63-1.33) | 0.91 (0.70-1.19) |
| Self-Harm hospital admissions | 13,263 | 24 | 24 | 1.00 (0.57-1.76) | 1.13 (0.75-1.70) |
| MI deaths | 3,533 | 3 | 4 | 0.75 (0.17-3.35) | 1.66 (0.56-4.90) |
| Suicide deaths | 689 | 2 | 4 | 0.50 (0.09-2.73) | 2.17 (0.63-7.50) |
| COPD deaths | 8,884 | 7 | 15 | 0.47 (0.19-1.15) | 0.72 (0.36-1.44) |
| All cause deaths | 52,637 | 22 | 43 | **0.51 (0.31-0.86)** | **0.60 (0.41-0.88)** |
|  |  | **NRT** |  |  |  |
| MI events | 20,599 | 161 | 104 | **1.55 (1.21-1.98)** | **1.78 (1.49-2.13)** |
| Self-harm events | 27,076 | 198 | 168 | 1.18 (0.96-1.45) | 1.15 (0.99-1.33) |
| Self-harm hospital admissions | 13,263 | 76 | 54 | 1.41 (0.99-2.00) | 1.06 (0.84-1.35) |
| MI deaths | 3,533 | 19 | 16 | 1.19 (0.61-2.31) | 0.87 (0.53-1.43) |
| Suicide deaths | 689 | 1 | 8 | 0.13 (0.02-1.0) | 0.40 (0.11-1.42) |
| COPD deaths | 8,884 | 75 | 70 | 1.07 (0.77-1.48) | 0.91 (0.71-1.17) |
| All cause deaths  Non-null findings are bolded | 52,637 | 241 | 252 | 0.96 (0.80-1.14) | 0.88 (0.77-1.01) |

*Matching on a maximum of four 30-day reference (ref) periods.
