# Supplementary material for: Risk of neuropsychiatric and cardiovascular adverse events following treatment with varenicline and nicotine replacement therapy in the UK Clinical Practice Research Datalink: a case–cross‐over study
Source: Addiction. 2020 Dec 14;116(6):1532–45. doi: 10.1111/add.15338 (PMC8246946; doi:10.1111/add.15338)
Supplement: Supplementary file 3 — Table S3 Odds ratios and 95% confidence intervals of exposure to varenicline and NRT using 180‐day risk and reference periods for specific adverse events. [file ADD-116-1532-s003.docx]

**Table S3. Odds ratios and 95% confidence intervals of exposure to varenicline and NRT using 180-day risk and reference periods for specific adverse events.**

| Adverse event | Number of events | Number Exposed risk period  Not exposed ref period | Number Not exposed risk period Exposed ref period | OR (95% CI)  1:1 matching | OR (95% CI)  1:4 matching* |
| --- | --- | --- | --- | --- | --- |
|  |  | **Varenicline** |  |  |  |
| MI events | 18,610 | 200 | 164 | 1.22 (0.99-1.50) | **1.49 (1.27-1.74)** |
| Self-Harm events | 24,035 | 270 | 233 | 1.16 (0.97-1.38) | **1.46 (1.27-1.67)** |
| Self-Harm hospital admissions | 12,026 | 114 | 101 | 1.12 (0.86-1.48) | **1.27 (1.04-1.56)** |
| MI deaths | 3,316 | 14 | 17 | 0.82 (0.41-1.67) | 0.79 (0.46-1.34) |
| Suicide deaths | 654 | 10 | 4 | 2.50 (0.78-7.97) | 2.13 (0.96-4.76) |
| COPD deaths | 8,486 | 46 | 48 | 0.96 (0.64-1.44) | **0.68 (0.51-0.93)** |
| All cause deaths | 50,029 | 161 | 230 | **0.70 (0.57-0.86)** | **0.68 (0.59-0.80)** |
|  |  | **NRT** |  |  |  |
| MI events | 18,610 | 424 | 373 | 1.14 (0.99-1.31) | **1.25 (1.13-1.39)** |
| Self-harm events | 24,035 | 664 | 656 | 1.01 (0.91-1.13) | **1.29 (1.19-1.41)** |
| Self-harm hospital admissions | 12,026 | 262 | 275 | 0.95 (0.80-1.13) | 1.09 (0.96-1.24) |
| MI deaths | 3,316 | 71 | 50 | 1.42 (0.99-2.04) | **1.35 (1.03-1.76)** |
| Suicide deaths | 654 | 13 | 24 | 0.54 (0.28-1.06) | 0.87 (0.49-1.56) |
| COPD deaths | 8,486 | 243 | 240 | 1.01 (0.85-1.21) | 1.10 (0.96-1.26) |
| All cause deaths  Non-null findings are bolded | 50,029 | 892 | 875 | 1.01 (0.93-1.12) | 1.10 (1.03-1.18) |

*Matching on a maximum of four 180-day reference (ref) periods
